# Population-based tobacco treatment: study design of a randomized controlled trial

**DOI:** 10.1186/1471-2458-12-159

**Published:** 2012-03-06

**Authors:** Steven S Fu, Michelle van Ryn, Scott E Sherman, Diana J Burgess, Siamak Noorbaloochi, Barbara Clothier, Anne M Joseph

**Affiliations:** 1 VA HSR&D Center for Chronic Disease Outcomes Research, Minneapolis VA Health Care System, Minneapolis, MN, USA; 2 University of Minnesota Medical School, Department of Medicine, Minneapolis, MN, USA; 3 University of Minnesota Medical School, Department of Family Medicine and Community Health, Minneapolis, MN, USA; 4 VA New York Harbor Healthcare System, New York City, NY, USA; 5 New York University School of Medicine, Division of General Internal Medicine, New York City, NY, USA

**Keywords:** Smoking Cessation, Tobacco Cessation Products, Ethnic Groups, Minority Health

## Abstract

**Background:**

Most smokers do not receive comprehensive, evidence-based treatment for tobacco use that includes intensive behavioral counseling along with pharmacotherapy. Further, the use of proven, tobacco treatments is lower among minorities than among Whites. The primary objectives of this study are to: (1) Assess the effect of a proactive care intervention (PRO) on population-level smoking abstinence rates (i.e., abstinence among all smokers including those who use and do not utilize treatment) and on utilization of tobacco treatment compared to reactive/usual care (UC) among a diverse population of smokers, (2) Compare the effect of PRO on population-level smoking abstinence rates and utilization of tobacco treatments between African American and White smokers, and (3) Determine the cost-effectiveness of the proactive care intervention.

**Methods/Design:**

This prospective randomized controlled trial identifies a population-based sample of current smokers from the Department of Veterans Affairs (VA) electronic medical record health factor dataset. The proactive care intervention combines: (1) proactive outreach and (2) offer of choice of smoking cessation services (telephone or face-to-face). Proactive outreach includes mailed invitation materials followed by an outreach call that encourages smokers to seek treatment with choice of services. Proactive care participants who choose telephone care receive VA telephone counseling and access to pharmacotherapy. Proactive care participants who choose face-to-face care are referred to their VA facility's smoking cessation clinic. Usual care participants have access to standard smoking cessation services from their VA facility (e.g., pharmacotherapy, smoking cessation clinic) and from their state telephone quitline. Baseline data is collected from VA administrative databases and participant surveys. Outcomes from both groups are collected 12 months post-randomization from participant surveys and from VA administrative databases. The primary outcome is self-reported smoking abstinence, which is assessed at the population-level (i.e., among those who utilize and those who do not utilize tobacco treatment). Primary analyses will follow intention-to-treat methodology.

**Discussion:**

This randomized trial is testing proactive outreach strategies offering choice of smoking cessation services, an innovation that if proven effective and cost-effective, will transform the way tobacco treatment is delivered. National dissemination of proactive treatment strategies could dramatically reduce tobacco-related morbidity, mortality, and health care costs.

**Clinical trials registration:**

ClinicalTrials.gov: NCT00608426.

## Background

Boosting utilization of evidence-based tobacco treatments and eliminating tobacco-related health disparities are top national tobacco control priorities [1]. Currently, tobacco cessation treatment relies on reactive treatment approaches that require smokers to either initiate treatment or to have a clinical encounter in which the provider has the time, initiative, and capacity to offer and deliver smoking cessation care. As a result, the majority of smokers do not receive evidence-based treatments (i.e., pharmacotherapy and/or behavioral counseling) that have demonstrated effectiveness for smoking cessation [2-4].

The population impact of tobacco treatment relies on exposure to treatment and is defined as the product of the rate of utilization of treatment (i.e., reach) and the efficacy of treatment (i.e., increase in smoking abstinence rates among those who utilize treatment) [2]. For example, a smoking cessation program may result in a 50% increase in quit rates among those utilizing the program but if only 2% of smokers utilize the program, the population impact would be 1%. Effective treatments, if rarely utilized, have negligible population impact; therefore, it is critical to maximize the reach of treatment.

In contrast to the two prevailing types of approaches which consist largely of either intensive individual-level intervention or traditional population-based intervention, a proactive-population-based approach combines the two and reaches out to all smokers in a given setting (for example, smokers in a large health care system) irrespective of level of motivation. Proactive treatment approaches have great potential to integrate individual and population-based perspectives and increase the population impact of tobacco cessation treatment by increasing the utilization of treatment with higher effectiveness. Despite their great potential to increase reach of more effective treatment, to date, there has been limited use of proactive treatment approaches for tobacco cessation.

In this paper, we describe the study design and methods of a prospective randomized controlled trial to determine the effects of a theory-driven intervention combining 1) proactive outreach with 2) choice of telephone care or face-to-face care for treatment of tobacco dependence (proactive care) compared to reactive care (usual care). This study overcomes prior limitations, namely the ability to identify populations of smokers in a health care system using the electronic medical record and the availability of telephone care that can be used to efficiently deliver intensive behavioral counseling and pharmacotherapy. The specific aims of this trial are to: 1) assess the effects of proactive care on population-level smoking abstinence rates and utilization of tobacco treatment compared to usual care among a diverse population of smokers who are enrolled in the Veterans Health Administration (VHA), the nation's largest integrated health care system, 2) compare the effects of proactive care on population-level smoking abstinence rates and utilization of tobacco treatment between African American and White smokers, and 3) determine the cost-effectiveness of the proactive care intervention.

## Methods/design

### Study design overview

**Figure 1:**
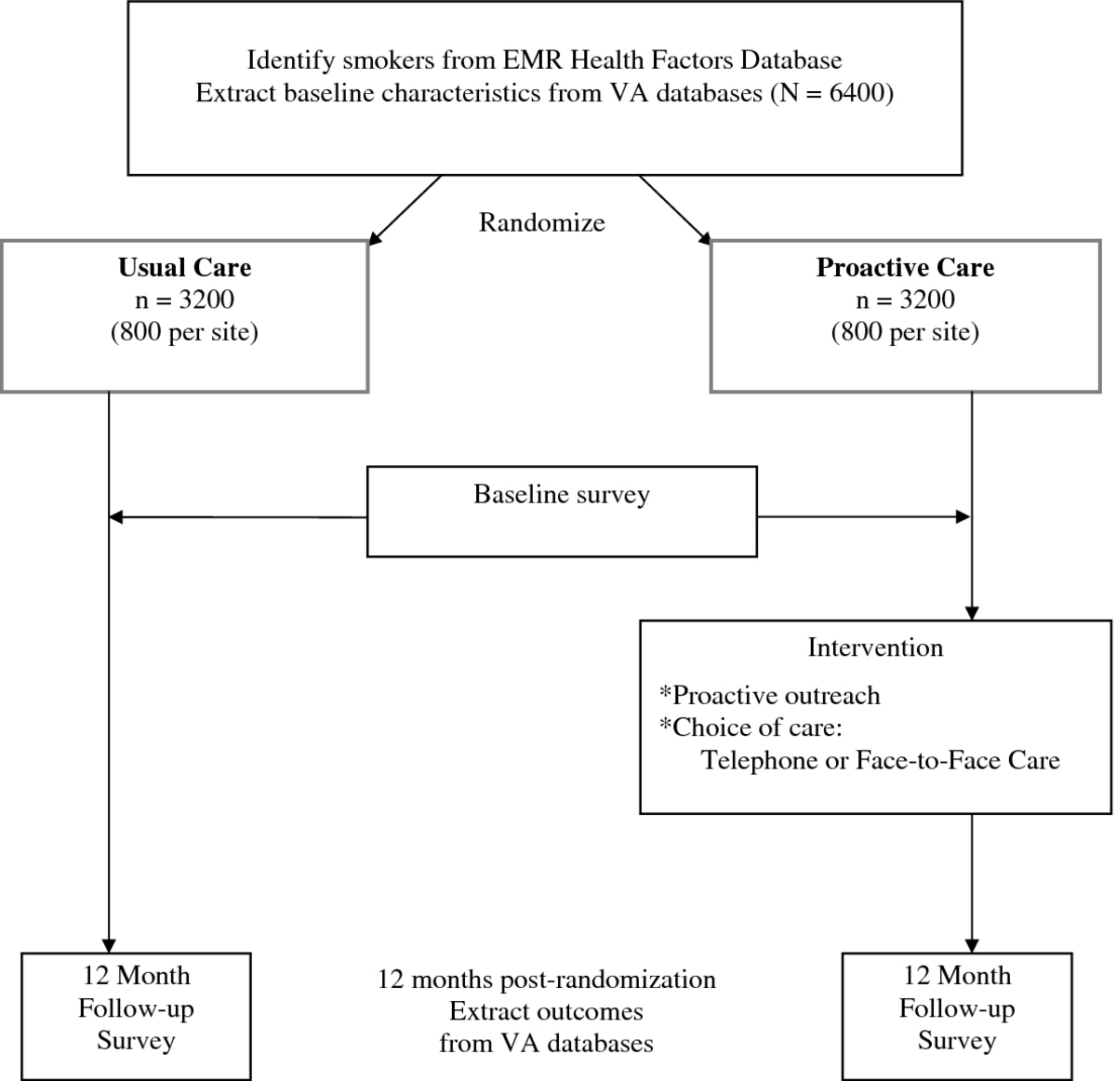
**Study Design and Overview**.

This study is funded by the Department of Veterans Affairs (VA) Health Services Research and Development (HSR&D) and is registered in clinicaltrials.gov (NCT00608426). The Veterans Victory over Tobacco Study is a randomized controlled trial that compares the effects of a proactive care intervention including proactive outreach and choice of telephone care or face-to-face care for tobacco dependence treatment compared to reactive care (usual care). This study considers the population impact of smoking cessation treatment. Therefore, all smokers are included regardless of their interest in quitting. The study design (Figure 1) is a complete block design with subsampling in which 1600 individuals from each of four study sites are randomized to one of two groups (total N = 6400). Hence, each site constitutes a block and within each block 800 are assigned to the intervention group and 800 are assigned to usual care. Blocking by site controls for variation in outcomes due to differences between sites such as differences in smoking cessation programs and related factors. The VA's electronic medical record (EMR) Health Factors Dataset is used to identify current smokers at the four study sites [4] and all smokers identified are randomized prior to contact. Baseline data is obtained from VA administrative databases and from a participant survey that is conducted immediately after randomization. Outcome data is being collected 12 months after randomization from VA administrative databases and from a follow-up survey.

### Study sites

This study is being conducted at four VA medical centers: the James A. Haley VA Medical Center (Tampa Bay, FL), the New York Harbor VA Medical Center (Manhattan, NY), the G.V. (Sonny) Montgomery VA Medical Center (Jackson, MS), and the Minneapolis VA Medical Center (Minneapolis, MN). This study received ethical approval from the institutional review boards of the Minneapolis VA Medical Center (FWA00001480), the New York Harbor VA Medical Center (FWA00001881), the G.V. Montgomery VA Medical Center (FWA00001598) and the University of Southern Florida (FWA00001669).

### Study participants

The sampling population for this study includes all Veterans ages 18 and older who are identified as current smokers from the VA's EMR Health Factors Dataset at each participating site. There are minimal exclusion criteria to maximize generalizability to the VA primary care population. However, any patient with an ICD9 diagnosis of dementia (i.e., 290.XX or 331.XX) or greater than 10 mental health clinic visits in the past year, determined from VA administrative data, is ineligible for study participation.

### Sampling procedures and strategy

The EMR Health Factors data are collected nationally using a clinical reminder process, which consists of automated requirements that providers must complete. The VA EMR tobacco use clinical reminder is primarily implemented in VA primary care clinics and information is stored in the Health Factors tables within the VA EMR databases [5]. The Health Factors tables are used to generate a list of Veterans at each participating facility who are identified as current smokers during a VA primary care visit in the past 3 months. Data extractions are run at each site every 3 months until the desired sample size at a given site for the two groups is accrued. A 3-month interval was chosen to ensure that we would have participants' most recent contact information.

### Intervention group: reactive/usual care

The usual care group receives access to tobacco treatment services from their local VA facility. Current VA national guidelines mandate screening for tobacco use and advising tobacco users to quit (i.e., brief counseling) and facilities are held accountable for their rates of screening and advice using performance measures, which are based on external audit of medical records. Treatment may be provided through primary care clinics and/or specialty tobacco treatment clinics. In addition, it is possible that participants may access help to quit smoking by calling their state quitline or the national quitline (1800QUITNOW). Treatment delivered by primary care providers may consist of brief counseling to quit and medication treatment. Pharmacotherapy is available in the form of nicotine patches, nicotine gum or bupropion. Medication costs in the VA are subsidized and may be free or a nominal co-pay of $9 per prescription is charged to Veteran depending upon their eligibility status. Participants may also be referred to a smoking cessation clinic from which they receive intensive counseling, which consists of behavioral and cognitive strategies to quit smoking. Behavioral counseling usually occurs in a group setting and participants are encouraged to attend at least 4 sessions, lasting 30-60 minutes, which are held once or twice per week. In addition, a limited amount of individual counseling is available. In sum, the cessation programs available at these 4 sites are representative of existing programs elsewhere in the VA and are comparable as to the availability of pharmacotherapy and face-to-face services.

### Intervention group: proactive care

The theory-driven proactive care intervention combines two main components: 1) proactive outreach (mailed invitation materials followed by telephone outreach) and 2) offer of choice of smoking cessation services (telephone care or face-to-face care), described below. Proactive outreach is coordinated by trained counselors at the Minneapolis VA Medical Center. Counselors have a BS or MS level degree in a health related field and received training in motivational interviewing and smoking cessation counseling from the California Smoker's Helpline and the study investigators. In addition to offering care, the mailed materials (invitation letter and brochure) and the proactive outreach call provides motivational enhancement to encourage participants to quit smoking and seek tobacco treatment. Participants who chose telephone care receive telephone counseling and care coordination from counselors at the Minneapolis VA. For proactive care participants who chose face-to-face care, the counselors arrange a referral to their local VA facility's smoking cessation program.

#### Intervention rationale

The intervention is based on Social Cognitive Theory (SCT) and the Stages of Change Model because choosing to utilize treatment and ultimately to quit is affected by a complicated web of both social and cognitive factors. SCT posits that cognitive processes largely regulate behavior and points to the importance of both cognitive (personal) and social environmental factors in determining behavior, which is highly relevant for tobacco cessation [6]. In addition, the intervention incorporates the Stages of Change model, which posits behavioral change occurs by moving along five distinct stages (precontemplation, contemplation, preparation, action and maintenance) [7]. Proactive care through the invitation letter and brochure, outreach call and telephone care cessation services is expected to largely address provider and psychosocial barriers to initiating care. The intervention eliminates provider barriers both by directly offering care and by providing the option of receiving such care in the convenience of their home over the phone. The intervention supplements provider treatment and does not preclude the patient from participating in services offered by the provider.

The intervention is expected to overcome psychosocial barriers through the following three mechanisms. First, motivational interviewing (MI) counseling is directed towards increasing both motivation to quit and self-efficacy as well as addressing environmental factors, such as social network norms. MI is a patient-centered counseling method whose main purpose is to help the participant explore and resolve ambivalence about a particular behavior change [8]. MI is an appropriate intervention for smokers at all levels of readiness to quit and entails asking the participant to talk about the pros and cons of the behavior and how the behavior fits in with his/her values and plans for the future. Important features of MI are an emphasis on collaboration with the participant, respect for his/her autonomy, and a goal of understanding the participant's point of view. The counselor avoids confrontation and takes advantage of many opportunities to enhance self-efficacy by pointing out and reinforcing patient strengths and successes. Recommendations for action are presented in the form of menus (i.e., the participant is provided choices).

Second, the intervention reduces barriers for smokers who have had or anticipate having negative medical care experiences and/or mistrust medical care by offering a program that does not require a medical care encounter. In addition, since MI is collaborative in nature, the therapeutic relationship is more like a partnership or companionship than expert/recipient roles. This process is expected to reduce fears of poor treatment, disrespect, or discomfort associated with being the lowest status person in an encounter.

Third, the invitation letter and the telephone counselors address the safety, efficacy, and functional benefits of pharmacotherapy. This is important because many smokers are misinformed and are unaware that pharmacotherapy can help alleviate withdrawal symptoms that have hindered their past cessation attempts [9,10]. Moreover, there is evidence that increasing knowledge about safety and efficacy increases rates of smokers' willingness to use pharmacotherapy [10], potentially increasing utilization rates.

#### Mailed invitation materials

Participants in the proactive care intervention group receive a personalized invitation letter and a brochure about the program. This invitation packet also contains a refrigerator magnet that includes the study logo and name, a toll-free phone number, and a statement, "You can quit. VA coaches can help." The letter and brochure describe the types of tobacco treatment services available from the VA to quit smoking, and to help them access treatment, offers the choice of telephone or face-to-face services. In addition, because targeted health communication messages that are specifically designed to be relevant for a particular subgroup have been effective at increasing smoking [11] as well as in changing other health-related behaviors, the letter includes targeted messages aimed at motivating smokers to seek treatment to quit smoking. These targeted messages were based on information gleaned from focus groups conducted with Veteran smokers. For example, the letter includes information addressing several barriers to minority and Veterans smokers' use of such as the lack of knowledge about the safety, efficacy and functional benefits of pharmacotherapy. The letter also addresses Veterans who are not ready to quit right now but who want more information about treatment options and "products that make it easier for smokers to quit."

#### Telephone outreach

Ten days after the mailed invitation packet is sent (or earlier for participants who respond to the letter), participants receive an outreach call from a counselor trained in motivational interviewing and smoking cessation treatment. Up to 6 contact attempts over a two week period are made at different times (i.e., morning, afternoon, evening) during the day. The purpose of the outreach call is to 1) describe the VA smoking cessation services available, 2) deliver motivational advice to quit smoking, and 3) encourage participants to participate in smoking cessation treatment.

#### Telephone care

Telephone care in this study combines proactive phone-based counseling with increased access to pharmacological therapy. The telephone care protocol is based on the TELESTOP study which demonstrated the benefits of telephone care compared to routine health care provider intervention for smoking cessation [12]. The TELESTOP protocol is an adaptation for Veterans of the evidence-based California Helpline protocol and uses a combination of motivational interviewing and cognitive-behavioral treatment for substance abuse [13]. Telephone counseling consists of 7 calls initiated by the counselor, scheduled in a manner to minimize the likelihood of relapse over a 2-month period (pre-quit, quit day, then 3 days, 1 week, 2 weeks, 1 months, and 2 months after the quit date). In the pre-quit session, a major portion of the counseling is spent promoting smokers' self-efficacy using motivational interviewing. Smokers are asked to identify situations in which it would be most difficult to refrain from smoking and to plan realistic coping strategies. During the follow-up calls, the emphasis is on successful implementation of effective coping strategies and relapse prevention. Participants who relapse during the program are encouraged to set new quit dates and are able to repeat the counseling protocol.

Consistent with national smoking cessation guidelines, counseling includes encouragement to use pharmacotherapy. The nicotine patch is recommended as the initial agent. Other forms of NRT (e.g., gum, lozenge), bupropion, or combinations of medications are recommended if the subjects have prior unsuccessful quit attempts using nicotine patch. Varenicline is considered second-line therapy in the VHA. Counselors facilitate access to pharmacotherapy by communicating with the participant's VHA primary care provider and/or smoking cessation clinician(s) at the local VHA facility. Telephone counselors enter an electronic progress note into the EMR to document the telephone counseling and the participant's preference for pharmacotherapy and designate the primary care provider and/or the smoking cessation clinician(s) as additional signers on the progress notes. The participant's VA providers are responsible for prescribing the smoking cessation medications as part of their routine clinical practice.

#### Face to face care

Face-to-face smoking cessation programs represent the standard of care for delivery of smoking cessation care in the VHA. For proactive care participants who chose face-to-face care, the counselors arrange a referral to their local VHA facility's smoking cessation clinic. Proactive care participants who chose face-to-face care receive the same care as usual care participants who are referred by their VHA provider.

### Data collection

Data collection occurs at baseline (i.e., at time of randomization) and at 12 months post-randomization. Data sources include survey data collected at baseline and at the twelve month follow up assessment, and VA administrative databases. Both the baseline survey and follow-up survey are 8-pages and are estimated to take the average participant 15 minutes to complete. The baseline survey consists of 79 questions and the follow-up survey includes 83 questions. The baseline survey mailing follows a modified Dillman protocol to maximize response rates and data quality (mail + postcard reminder + mail + mail) [14]. The 1^st ^survey mailing includes a cover letter describing the study, a $10 cash incentive, the self-administered survey instrument, a postage paid return envelope, and an informed consent statement describing the study. Approximately 10 days later, a reminder postcard is mailed to non-respondents. About two to three weeks after the postcard is mailed, a 2^nd ^survey, cover letter, pen, and postage paid return envelope is mailed to all participants who have not yet returned a survey. About two to three weeks after the 2^nd ^survey mailing, participants who do not return a mailed survey are mailed a shortened, 2-page survey, cover letter, and a postage paid return envelope.

The 12-month follow-up survey also follows a modified Dillman protocol. First, participants are sent a pre-letter explaining that they will be receiving a survey in the mail within a few days. A few days after the pre-letter, participants are mailed a survey. This mailing includes a cover letter, a $10 cash incentive, the follow-up survey, and a postage paid return envelope. A reminder post-card is mailed to non-respondents about 10 days after the 1st survey mailing. About two weeks later, non-respondents are mailed a second survey along with a cover letter, a pen, and a postage paid return envelope. Approximately two weeks later, anyone who has not responded to the survey is randomized to one of two methods of contact for the fifth and final contact: 1) mailed a shortened, 2-page follow-up survey via USPS first class mail along with a cover letter and a postage paid return envelope, or 2) telephone administration of the shortened, follow-up survey. Up to six telephone contact attempts are made at various times of the day. There are several reasons for the experiment. The first reason is to see if the additional cost of a telephone follow-up is an efficient method for increasing response rates. Also, there is movement toward surveys that use more than one mode of data collection, but it is not clear if it is the additional attempts that increase response rate, or the change in mode. This experiment will allow us to examine if changing the mode for a final attempt increases response rates, or if the mode of the final contact does not matter.

### Outcome measures

The primary outcome is self-reported smoking abstinence of 6 months duration, irrespective of treatment utilization (i.e., smoking abstinence among all smokers including those who use and those who do not use treatment), measured at 12 months, determined from the follow-up survey 12 months after randomization. Following the Society for Research on Nicotine and Tobacco (SRNT) Measures Workgroup Recommendations, the primary outcome is defined as having not smoked at least part of a cigarette on each of 7 consecutive days and having not smoked at least once on a weekend day over 2 consecutive weekends in the past 6 months [15]. Secondary measures of abstinence include self-reported 7-day point prevalence and 30-day point prevalence, defined as having not smoked a part of a cigarette in the past 7 days and as having not smoked a part of a cigarette in the past 30 days, respectively.

Other outcomes for this study comprise utilization and initiation of tobacco cessation treatment (from any source, including supplemental care from outside the VA) during the 12 month follow-up, specifically: 1) initiation of combined intensive behavioral counseling and medication treatment, 2) initiation of intensive behavioral counseling, and 3) initiation of medication treatment. Since some smokers may seek treatment from non-VA providers due to the proactive strategies tested, the secondary outcomes are primarily assessed using self-report from the 12-month follow-up survey. Initiation of medication treatment is defined as using one or more tobacco dependence medications (e.g., NRT and bupropion) in the 12-month follow-up period (from any source, VA and non-VA). Initiation of intensive counseling is defined as the completion of at least one call from a telephone counseling program or making at least one visit to a smoking cessation program in the 12-month follow-up period (from any source, VA and non-VA). In addition, we are assessing VA tobacco treatment utilization using VA administrative data--pharmacy dispensing records and visits to smoking cessation clinics

### Potential moderators and confounders

#### Background factors

The following patient demographics are assessed in the survey: race, ethnicity, marital status, education, annual household income, and employment status. Alcohol consumption is assessed using the AUDIT-C [16]. VA administrative data sources are used to provide information on age, sex, and co-morbid conditions (e.g., ICD-9 codes).

#### Smoking behavior

Information regarding smoking history such as age of initiation, duration of smoking, previous quit attempts and prior use of tobacco treatment is collected using standard questions from the California Tobacco Survey [17] and the CDC Behavioral Risk Factor Surveillance Survey [18]. Nicotine dependence is assessed with the two-question Heaviness of Smoking Index: cigarettes per day and time to first cigarette after waking [19].

### Potential mediators

#### Provider factors

The provider factors examined include provider behavior related to delivery of smoking cessation care and communication style. Standard tobacco performance measures assess participants' receipt of smoking cessation advice, counseling and treatment from their VA primary care provider [20]. Questions from the Commonwealth Fund Survey are used to assess provider communication [21].

#### Cognitive factors

The cognitive factors examined in this study include motivation to quit, self-efficacy, attitudes toward NRT, mastery, and smoking stigma. Self-efficacy to quit and motivation to quit are assessed with a global measure of self-efficacy to quit [22], three self-efficacy subscales (situational self-efficacy which includes emotional and social self-efficacy and skill self-efficacy) [23] and the readiness to quit ladder [24,25]. Beliefs towards smoking cessation medications is assessed using the 12-item attitudes towards nicotine replacement therapy scale (ANRT-12) [26]. Mastery is measured using a standard 7-item questionnaire assessing the control one feels over one's life [27]. Smoking stigma, the concept that one feels stigmatized due to their smoking behavior, has been adapted from the Mental Health Consumers' Experience of Stigma [28].

#### Discrimination

In this study discrimination in the health care setting is assessed using the Physician Bias and Interpersonal Cultural Competence Measures Scale [21] which consists of 3 questions asking about being treated with respect by the doctor, the doctor's understanding of the participant's background and values, and feeling like the doctor looks down on the participant's way of life. Health care discrimination is also measured with a general question asking if the patient feels they have been discriminated against in a health care situation for any reason [29]. Day to day perceived discrimination is measured using nine questions that ask about the frequency of exposure to experiences of discrimination such as being treated with less courtesy, less respect, or being harassed [30].

#### Social environment

Participants' self-report their level of satisfaction with smoking cessation care from the VA on the follow-up survey [31]. Characteristics of the environment that make it difficult for them to participate in smoking cessation care from the VA are also being assessed (e.g., lack of transportation, and distance to site) [13]. Characteristics of the patient's social network assessed in the survey include: subjective norms related to smoking, smoking habits of friends and family, and home smoking rules.

### Analytic overview

With our proposed sample size (3200 per intervention group), random assignment can be expected to create two groups that are balanced with respect to observed and unobserved baseline characteristics. However, due to possible differential non-response among the two groups this balance may be missing between survey respondents within the two groups. To assess such imbalance, the respondents in the two groups will be compared with respect to key elements of the conceptual model and baseline measures a priori known to be related to smoking cessation and treatment utilization. Balance of the two groups will be tested using Mantel-Haenszel *χ*^2 ^tests for categorical variables which accounts for stratification by site and appropriate parametric tests (e.g. Blocked Anova F-tests) or nonparametric tests (e.g. Wilcoxon rank-sum test) for continuous variables. If the two groups are found to differ with respect to these relevant pre-intervention variables, then these variables will be included as covariates in the following analyses. These analyses and related comparisons of responders and non-responders will shed light on potential response bias issues. A usual practice in is to treat non-respondents at follow-up as continuing smokers. This practice is considered to be a conservative assumption; however, if usual care has significantly lower response rates to the follow-up survey than proactive care then this approach would favor the intervention. To address potential response bias issues, we will use standard statistical methodologies and implement propensity-based and imputation methods with imputation of smoking status or treatment utilization at follow-up. For VA treatment utilization outcomes, non-response is not an issue because we will obtain information from VA administrative databases on all randomized individuals.

The primary analysis will be by intention-to-treat. Since population-level smoking abstinence rates are expected to be small, z-tests comparing the strata-weighted differences between the two groups are less appropriate than comparisons based on odds ratios. The study design is a randomized complete block design, however, the patient populations within site vary and hence the proportion of the site population included in the study varies across site. Different intra-block (stratified) logistic analyses have been devised for similar survey designs for sampling from finite populations [32,33]. Herein these survey design-based logistic analyses are referred to as survey-logistic regressions. We will use survey-logistic regression methods to estimate and test intervention effects and racial/ethnic differences in the intervention effects on both smoking abstinence (e.g., 6-month prolonged abstinence, 7-day and 30-day point prevalence abstinence) and treatment utilization rates. These regression models will take into account the design stratification and the relative stratification populations. The models also will include any relevant covariates to adjust for confounding, if warranted. In this event we will use cross validation methods and standard model diagnostic methods to identify a well fitting model. Inference with respect to the impact of the intervention will be based on point estimates of the odds ratio for the effect of proactive outreach on the outcome relative to usual care, along with 95% confidence intervals and corresponding standard test results.

### Sample size and power analysis

The power analysis assumes independent samples in the two groups from the four sites and considers a type one error rate of 0.05. A meaningful difference, from a public health perspective, is for PRO to produce a 2% increase in population-level smoking abstinence rates compared to UC. Using a base rate of 3% to estimate the sample size for this study, this study requires 1500 participants per intervention group (or total N = 3000) to have at least 80% power to detect a 2% or greater absolute difference in population-level smoking abstinence rates between groups. In addition, this study is well powered (> 90%) to detect clinically meaningful differences of 6% in treatment utilization rates using the most conservative base rate assumption of 50%. In order to achieve 3000 completed follow-up surveys from participants, we estimated that we need to include 6400 participants who will be sent the baseline survey. Among the 6400 participants who are mailed a baseline survey, we estimate approximately 5120 participants (20% exclusion rate due to subject refusal and misclassification of cigarette smoking status) will be sent a follow-up survey. We estimate a 60% response rate to the follow-up survey, which will result in about 3000 completed follow-up surveys.

## Discussion

In general, population-based interventions lead to higher utilization rates but may have lower efficacy because they are less intensive (e.g., mass mailings of self-help materials) whereas individual-level interventions might have higher efficacy but lower utilization rates. Intensive individual-level interventions include clinic-based treatment and generally involve reactively recruited smokers (i.e. individuals who are ready to quit and seek out assistance). While clinic-based treatments yield high long-term quit rates, on the order of 20-30%, they are also known to have the low utilization rates [34]. For example, only 1-10% of smokers in managed care organizations use the state-of-the science smoking cessation clinics the systems offer [2,35,36]. In spite of national guidelines, only about 1 in 3 smokers who visited a health care provider in the past year used a tobacco dependence treatment during a quit attempt [4]. Over 70% of smokers say they want to quit, leaving a huge gap between interest in quitting and use of treatment services [34].

The Veterans Victory Over Tobacco Project bridges individual and population-based perspectives to increase the population impact of tobacco treatment by increasing utilization of more effective programs. The proactive care intervention evaluated in this study is hypothesized to have a greater population impact because it combines strategies that can 1) achieve wide reach and increase utilization of treatment (proactive outreach), and 2) increase the effectiveness of treatment by efficiently delivering intensive behavioral and pharmacologic treatments (option of telephone care).

Previously, proactive treatment approaches have been limited by the inability to efficiently identify current smokers in the general population and by the reliance on in-person smoking cessation counseling. One barrier to the use of proactive treatment approaches is difficulty identifying current smokers in the general population. A second issue involves the availability of an intervention that is both effective and feasible to administer using a proactive approach. Interventions that have taken a proactive, population-based approach usually have delivered less intensive interventions such as mailings of self-help quitting manuals [37,38]. However, a few recent studies have capitalized on the availability of validated telephone counseling protocols to provide intensive behavioral counseling with pharmacotherapy [39,40] on a large-scale, in a way that would be feasible for a proactive treatment approach.

For example, two studies identified smokers using pharmacy databases from large health plans, based on tracking individuals who had recently filled a prescription for a tobacco dependence medication. To offer telephone counseling as an adjunct to pharmacotherapy, smokers in the first study received proactive outreach which involved a mailed letter and a telephone call. Proactive outreach resulted in 31% (205/663) of smokers utilizing telephone counseling [39]. Similarly, in the second study, a proactive phone call (no letter) resulted in 21% of smokers utilizing telephone counseling [40]. In addition, a community survey assessed adult smokers' response to direct telemarketing of smoking cessation interventions and found that almost half would utilize a "we-call-you" telephone counseling service [41]. The proposed study extends this research because all smokers are being identified from the electronic medical record, not just those smokers who have already received pharmacologic treatment, and the proactive care interventions offers a choice of telephone or face-to-face smoking cessation services.

The strength of this project is the use of a population-based approach to evaluate the effectiveness of proactive outreach and choice of care on population-level quit rates. All smokers, regardless of motivational level to quit smoking are included, and therefore, findings will have greater generalizability than traditional clinical trials. However, the intervention combines two main components (proactive outreach and the option of telephone care); therefore, if it has a positive effect on population-level smoking abstinence rates, it will be difficult to determine which component(s) are responsible. However, the intervention components integrate individual and population-based approaches and so are important to evaluate together.

In conclusion, this study is significant because it has the potential to transform and improve the effectiveness of the delivery of tobacco treatment. National guidelines have made it a priority to increase access to tobacco treatment as part of a population-based approach to tobacco cessation [3]. If proven to be effective and acceptably low-cost, national dissemination of proactive treatment approaches would have potential to dramatically reduce tobacco-related morbidity, mortality, and health care costs for the nation.

## Competing interests

The authors declare that they have no competing interests.

## Authors' contributions

SSF and AMJ conceived the study design and drafted the study protocol. MVR, SES, DJB, SN, BC helped to draft the study protocol. All authors provided critical review of the study protocol and approved the final manuscript.

## Pre-publication history

The pre-publication history for this paper can be accessed here:

http://www.biomedcentral.com/1471-2458/12/159/prepub

## References

[B1] Fiore MC Croyle RT Curry SJ Cutler CM Davis RM Gordon C Healton C Koh HK Orleans CT Richling D Satcher D Seffrin J Williams C Williams LN Keller PA Baker TB Preventing 3 million premature deaths and helping 5 million smokers quit: a national action plan for tobacco cessation Am J Public Health 2004 94 205 210 10.2105/AJPH.94.2.205 14759928 PMC1448229

[B2] Abrams DB Orleans CT Niaura RS Goldstein MG Prochaska JO Integrating individual and public health perspectives for tobacco treatment under managed health care: a combined stepped-care and matching model Ann Behav Med 1996 18 290 304 10.1007/BF02895291 18425675

[B3] Sherman SE Talcot W (Co-Chairs) Veterans Health Administration/Department of Defense Clinical Practice Guideline on Management of Tobacco Use 2004 http://www.oqp.med.va.gov/cpg/TUC3/tuc_base.htm. Accessed 2/1/2005

[B4] Cokkinedes VE Halpern MT Barbeau EM Ward E Thun MJ Racial and Ethnic Disparities in Smoking Cessation Interventions Am J Prev Med 2008 34 404 412 10.1016/j.amepre.2008.02.003 18407007

[B5] McGinnis KA Brandt CA Skanderson M Justice AC Shahrir S Butt AA Brown ST Freiberg MS Gibert CL Goetz MB Kim JW Pisani MA Rimland D Rodriguez-Barradas MC Sico JJ Tindle HA Crothers K Validating Smoking Data From the Veteran's Affairs Health Factors Dataset, an Electronic Data Source Nicotine Tob Res 2011 13 1233 1239 10.1093/ntr/ntr206 21911825 PMC3223583

[B6] Bandura A Social foundations of thought and action: A social cognitive theory 1986 Englewood Cliffs, NJ: Prentice-Hall

[B7] Prochaska JO DiClemente CC Stages and process of self-change of smoking: Toward an integrative model of change J Consult Clin Psychol 1983 51 390 395 6863699 10.1037//0022-006x.51.3.390

[B8] Miller WR Rollnick S Motivational interviewing: Preparing people for change 2002 New York: Guilford Press

[B9] Fu SS Burgess D van Ryn M Hatsukami DK Solomon J Joseph AM Views on smoking cessation methods in ethnic minority communities: a qualitative investigation Prev Med 2007 44 235 240 10.1016/j.ypmed.2006.11.002 17175016

[B10] Bansal MA Cummings KM Hyland A Giovino GA Stop-smoking medications: who uses them, who misuses them, and who is misinformed about them? Nicotine Tob Res 2004 6 Suppl 3 S303 S310 15799593 10.1080/14622200412331320707

[B11] Strecher VJ Computer-tailored smoking cessation materials: a review and discussion Patient Educ Couns 1999 36 107 117 10.1016/S0738-3991(98)00128-1 10223016

[B12] An LC Zhu SH Nelson DB Arikian NJ Nugent S Partin MR Joseph AM Benefits of telephone care over primary care for smoking cessation: a randomized trial Arch Intern Med 2006 166 536 542 10.1001/archinte.166.5.536 16534040

[B13] Zhu SH Anderson CM Tedeschi GJ Rosbrook B Johnson CE Byrd M Gutiérrez-Terrell E Evidence of real-world effectiveness of a telephone quitline for smokers N Engl J Med 2002 347 1087 1093 10.1056/NEJMsa020660 12362011

[B14] Dillman DA Mail and Internet Surveys: The Tailored Design Method 2000 New York, NY: John Wiley & Sons Inc.

[B15] Hughes JR Keely JP Niaura RS Ossip-Klein DJ Richmond RL Swan GE Measures of abstinence in clinical trials: issues and recommendations Nicotine Tob Res 2003 5 13 25 12745503

[B16] Bush K Kivlahan DR McDonell MB Fihn SD Bradley KA The AUDIT alcohol consumption questions (AUDIT-C): an effective brief screening test for problem drinking. Ambulatory Care Quality Improvement Project (ACQUIP). Alcohol Use Disorders Identification Test Arch Intern Med 1998 158 1789 1795 10.1001/archinte.158.16.1789 9738608

[B17] California Adult Tobacco Survey http://www.cdph.ca.gov/data/surveys/Pages/CaliforniaTobaccoSurveys.aspx Accessed 12/8/2011

[B18] Behavioral Risk Factor Surveillance System Questionnaire http://www.cdc.gov/brfss/questionnaires/english.htm Accessed 12/8/2011

[B19] Heatherton TF Kozlowski LT Frecker RC Rickert W Robinson J Measuring the heaviness of smoking: using self-reported time to the first cigarette of the day and number of cigarettes smoked per day Br J Addict 1989 84 791 799 10.1111/j.1360-0443.1989.tb03059.x 2758152

[B20] Davis RM Healthcare report cards and tobacco measures Tob Control 1997 6 Suppl S70 S77 9396129

[B21] Johnson RL Saha S Arbelaez JJ Beach MC Cooper LA Racial and ethnic differences in patient perceptions of bias and cultural competence in health care J Gen Intern Med 2004 19 101 110 15009789 10.1111/j.1525-1497.2004.30262.x PMC1492144

[B22] Baldwin AS Rothman AJ Hertel AW Linde JA Jeffery RW Finch EA Lando HA Specifying the determinants of the initiation and maintenance of behavior change: an examination of self-efficacy, satisfaction, and smoking cessation Health Psychol 2006 25 626 634 17014280 10.1037/0278-6133.25.5.626

[B23] Dijkstra A De Vries H Self-efficacy expectations with regard to different tasks in smoking cessation Psychol Health 2000 15 501 511 10.1080/08870440008402009

[B24] Abrams DB Niaura RS Brown RA Emmons KM Goldstein MG Monti PM The Tobacco Dependence Treatment Handbook 2003 New York: The Guilford Press

[B25] Biener L Abrams DB The Contemplation Ladder: validation of a measure of readiness to consider smoking cessation Health Psychol 1991 10 360 365 1935872 10.1037//0278-6133.10.5.360

[B26] Etter JF Perneger TV Attitudes toward nicotine replacement therapy in smokers and ex-smokers in the general public Clin Pharmacol Ther 2001 69 175 183 10.1067/mcp.2001.113722 11240982

[B27] Pearlin LI Schooler C The structure of coping J Health Soc Behav 1978 19 2 21 10.2307/2136319 649936

[B28] Wahl OF Mental health consumers' experience of stigma Schizophr Bull 1999 25 467 478 10478782 10.1093/oxfordjournals.schbul.a033394

[B29] Trivedi AN Ayanian JZ Perceived discrimination and use of preventive health services J Gen Intern Med 2006 21 553 558 10.1111/j.1525-1497.2006.00413.x 16808735 PMC1924636

[B30] Kessler RC Mickelson KD Williams DR The prevalence, distribution, and mental health correlates of perceived discrimination in the United States J Health Soc Behav 1999 40 208 230 10.2307/2676349 10513145

[B31] Ware JE Jr Snyder MK Wright WR Davies AR Defining and measuring patient satisfaction with medical care Eval Program Plann 1983 6 247 263 10.1016/0149-7189(83)90005-8 10267253

[B32] Lehtonen R Pahkinen E Practical Methods for Design and Analysis of Complex Surveys 1995 Chichester: John Wiley & Sons, Inc.

[B33] Binder DA Roberts GR Skinner C, Chambers R Design-based and Model-based Methods for Estimating Model Parameters Analysis of Survey Data 2003 Chichester, UK: Wiley 29 48

[B34] Fiore MC Jaen CJ Baker TB Treating Tobacco Use and Dependence: 2008 Update. Clinical Practice Guideline 2008 Rockville, MD: U.S. Department of Health and Human Service, Public Health Service

[B35] Sherman SE Yano EM Lanto AB Simon BF Rubenstein LV Smokers' interest in quitting and services received: using practice information to plan quality improvement and policy for smoking cessation Am J Med Qual 2005 20 33 39 10.1177/1062860604273776 15782753

[B36] Lichtenstein E Hollis J Patient referral to a smoking cessation program: who follows through? J Fam Pract 1992 34 739 744 1593248

[B37] Prochaska JO Velicer WF Fava JL Rossi JS Tsoh JY Evaluating a population-based recruitment approach and a stage-based expert system intervention for smoking cessation Addict Behav 2001 26 583 602 10.1016/S0306-4603(00)00151-9 11456079

[B38] Curry SJ McBride C Grothaus LC Louie D Wagner EH A randomized trial of self-help materials, personalized feedback, and telephone counseling with nonvolunteer smokers J Consult Clin Psychol 1995 63 1005 1014 8543703 10.1037//0022-006x.63.6.1005

[B39] Boyle RG Solberg LI Asche SE Boucher JL Pronk NP Jensen CJ Offering telephone counseling to smokers using pharmacotherapy Nicotine Tob Res 2005 7 Suppl 1 S19 S27 16036266 10.1080/14622200500078048

[B40] Holtrop JS Wadland WC Vansen S Weismantel D Fadel H Recruiting health plan members receiving pharmacotherapy into smoking cessation counseling Am J Manag Care 2005 11 501 507 16095436

[B41] Paul CL Wiggers J Daly JB Green S Walsh RA Knight J Girgis A Direct telemarketing of smoking cessation interventions: will smokers take the call? Addiction 2004 99 907 913 10.1111/j.1360-0443.2004.00773.x 15200586

